# The effects of acute and prolonged CRAM supplementation on reaction time and subjective measures of focus and alertness in healthy college students

**DOI:** 10.1186/1550-2783-7-39

**Published:** 2010-12-15

**Authors:** Jay R Hoffman, Nicholas A Ratamess, Adam Gonzalez, Noah A Beller, Mattan W Hoffman, Mark Olson, Martin Purpura, Ralf Jäger

**Affiliations:** 1 The University of Central Florida, Orlando, FL 32816-1250, USA; 2 The College of New Jersey, Ewing, NJ 08628, USA; 3 MRM, 2665 Vista Pacific Dr., Oceanside, CA 92056, USA; 4 Increnovo LLC, 2138 E. Lafayette Pl, Milwaukee, WI 53202, USA

## Abstract

**Background:**

The purpose of this study was to examine the effect of acute and prolonged (4-weeks) ingestion of a supplement designed to improve reaction time and subjective measures of alertness, energy, fatigue, and focus compared to placebo.

**Methods:**

Nineteen physically-active subjects (17 men and 2 women) were randomly assigned to a group that either consumed a supplement (21.1 ± 0.6 years; body mass: 80.6 ± 9.4 kg) or placebo (21.3 ± 0.8 years; body mass: 83.4 ± 18.5 kg). During the initial testing session (T1), subjects were provided 1.5 g of the supplement (CRAM; α-glycerophosphocholine, choline bitartrate, phosphatidylserine, vitamins B3, B6, and B12, folic acid, L-tyrosine, anhydrous caffeine, acetyl-L-carnitine, and naringin) or a placebo (PL), and rested quietly for 10-minutes before completing a questionnaire on subjective feelings of energy, fatigue, alertness and focus (PRE). Subjects then performed a 4-minute quickness and reaction test followed by a 10-min bout of exhaustive exercise. The questionnaire and reaction testing sequence was then repeated (POST). Subjects reported back to the lab (T2) following 4-weeks of supplementation and repeated the testing sequence.

**Results:**

Reaction time significantly declined (p = 0.050) between PRE and POST at T1 in subjects consuming PL, while subjects under CRAM supplementation were able to maintain (p = 0.114) their performance. Significant performance declines were seen in both groups from PRE to POST at T2. Elevations in fatigue were seen for CRAM at both T1 and T2 (p = 0.001 and p = 0.000, respectively), but only at T2 for PL (p = 0.029). Subjects in CRAM maintained focus between PRE and POST during both T1 and T2 trials (p = 0.152 and p = 0.082, respectively), whereas significant declines in focus were observed between PRE and POST in PL at both trials (p = 0.037 and p = 0.014, respectively). No difference in alertness was seen at T1 between PRE and POST for CRAM (p = 0.083), but a significant decline was recorded at T2 (p = 0.005). Alertness was significantly lower at POST at both T1 and T2 for PL (p = 0.040 and p = 0.33, respectively). No differences in any of these subjective measures were seen between the groups at any time point.

**Conclusion:**

Results indicate that acute ingestion of CRAM can maintain reaction time, and subjective feelings of focus and alertness to both visual and auditory stimuli in healthy college students following exhaustive exercise. However, some habituation may occur following 4-weeks of supplementation.

## Background

Phosphatidylcholine is a phospholipid that is a major constituent of all cellular membranes and is necessary for normal cellular function. It has an important role in the membrane's structural integrity and plays a vital role in supporting membrane expansion as the cells grow [1]. Phosphatidylcholine has a number of important physiological functions in the liver, gastrointestinal tract, kidneys, brain and in neuromuscular signal transmission. It is the latter role that may have a potential ergogenic effect during exercise. Choline is an essential nutrient that has an important function in synthesis of the neurotransmitter acetylcholine.

Neurons are unable to synthesize choline and rely on dietary intake to insure sufficient acetylcholine production [2]. Acetylcholine is critical for many physiological functions and any deficiency could result in a multitude of physiological problems. One of the more interesting findings has been the benefit that choline supplementation has had on memory and cognition improvements [3-6]. The importance of enhancing neurotransmitter function has interesting implications for athletic performance. Exercise that reduces plasma choline concentrations (i.e. marathon running) has been suggested to benefit from choline supplementation [7,8]. However, support for this hypothesis has been lacking [9,10]. This may be related to the inability of prolonged exercise to deplete plasma choline concentrations to levels that result in performance decrements [9,10]. However, if choline can improve neurotransmitter concentration then it stands to reason that it may have a potential ergogenic role in athletic events that involve power performance and the ability to react to external stimuli, even during events that plasma choline concentrations are normal. Choline, provided as phosphatidylcholine, is 12-fold more effective than inorganic choline salts in increasing serum concentrations and maintaining elevated concentrations for a longer duration (12 hours versus 30 minutes) [11,12]. Thus, most supplement studies will provide choline as phosphatidylcholine or L-Alpha Glycerylphosphorylcholine (alpha-GPC), a water-soluble form lacking the hydrophobic tail groups. In addition to being an excellent source of choline, acute alpha-GPC supplementation has been shown to augment growth hormone response to resistance exercise [13].

Phosphatidylserine is also a phospholipid that is incorporated into the membrane of organs with high metabolic activity such as brain, heart, lung, liver and skeletal muscle [14,15]. Several studies have demonstrated that phosphatidylserine may reduce inflammation ([16,17] and act as an antioxidant [18,19]. These properties have led to additional investigations on the ability of phosphatidylserine to enhance recovery from exercise. A 750 mg dosing of phosphatidylserine for 7 days prior to an acute bout of exhaustive exercise resulted in significant improvements in sprint performance [20] and an increase in time to exhaustion during intermittent cycling exercise [21]. Starks and colleagues [15] reported a lowered stress response to moderate intensity cycling exercise (65% - 85% VO_2_max) following 10 days of supplemention with 600 mg of phosphatidylserine, reflected by a reduced cortisol response to exercise. However, Kingsley and colleagues [22] were unable to support an improved recovery in individuals performing an acute bout of eccentric exercise (downhill running).

Investigations examining the combination of these phospholipids on enhancing exercise performance are limited, especially in exercise involving power performance and reaction time. Thus, the purpose of this study was to examine the acute effect of a low-dose combination of these phospholipids on reaction time, anaerobic power and subjective measures of alertness, energy, fatigue, and focus in health college students.

## Methods

### Subjects

Nineteen subjects (17 men and 2 women) volunteered for this study. Following an explanation of all procedures, risks, and benefits, each subject gave their informed consent to participate in this study. The Institutional Review Board of the College approved the research protocol. Subjects were not permitted to use any additional nutritional supplements throughout the experimental period. Screening for supplement use was accomplished via a health history questionnaire completed by the subjects during recruitment. All subjects were recreationally active for at least three months prior to the investigation.

Subjects were randomly assigned to a group that either consumed the supplement (21.1 ± 0.6 years; height: 180.2 ± 6.1 cm; body mass: 80.6 ± 9.4 kg; body fat %: 11.3 ± 6.9%) or a placebo (21.3 ± 0.8 years; height: 181.3 ± 10.2 cm; body mass: 83.4 ± 18.5 kg; body fat %: 14.9 ± 7.7%). The study was conducted in a double-blind format.

### Study Protocol

Subjects reported to the Human Performance Laboratory on two separate occasions (T1 and T2) for testing. Each testing session was separated by 4-weeks. Subjects were instructed to refrain from consuming any caffeine products on the day of each testing session and from performing any strenuous physical activity for the previous 12 hours. In addition, subjects were instructed not to eat or drink for 3 hours prior to each trial. Following a 10-min resting period subjects were provided with either the supplement (CRAM) or the placebo (PL). Subjects then rested quietly for 10-minutes prior to completing a 9-question survey ascertaining their subjective feelings for that moment relating to alertness, energy, fatigue, focus, and well-being. Following the survey subjects performed a 4-min reaction test (PRE). Upon completion of the reaction test subjects performed an additional 10-min of exhaustive exercise before repeating the survey and reaction test (POST). Prior to the onset of the initial reaction test all subjects performed a 5-minute warm-up on a cycle ergometer and a standardized movement-specific warm-up that included a short sprint, side shuffles and a backwards run. The exhaustive exercise period consisted of a 30-second Wingate Anaerobic Power test, maximal number of push-ups for one-minute and the maximal number of sit-ups within a one-minute period.

**Figure 1:**
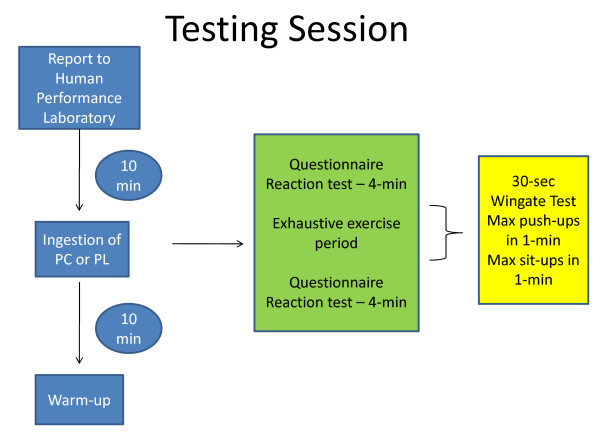
**Testing Session**.

To examine the effect of prolonged supplementation subjects continued to consume either the supplement or placebo every day for four consecutive weeks. At the end of 4-weeks of supplementation subjects reported back to the Human Performance Laboratory and repeated the testing protocol. The testing sequence is depicted in Figure 1.

#### Reaction Test

Reaction time was assessed using the Makoto testing device (Makoto USA, Centennial CO). The Makoto device is triangular in shape that is eight feet from base to apex. It consists of three steel towers that are six feet high. Each tower contains ten targets. For each test the subject stood in the middle of the triangle and faced one of the towers with the other two in his/her peripheral vision. The reaction test began with a loud auditory stimulus. During the next four minutes subjects were required to react to both a visual (targets light up) and auditory (loud gong) stimulus. As the gong sounded and the light on the target lit up the subject was required to lunge and make contact with the target with their hands. Subjects had to make contact to the target prior to the light and sound stopping. If the subject made contact with the target within the required time it was registered as a 'hit'. Subjects were required to make as many contacts as possible within the 4-min period.

All subjects completed familiarization sessions on the Makoto device prior to entering the study. To enroll in the study subjects were required to achieve 65% success rate at level 8 on the Makoto device for two consecutive sessions. Subjects performed on average 4.1 ± 0.8 familiarization sessions. To maintain technique and skill on the Makoto device during the 4-week supplementation period subjects continued to perform a single 4-minute trial once per week.

#### Anaerobic Power Measure

To quantify anaerobic power performance all subjects performed a modified Wingate Anaerobic Power test (Lode Excalibur, Groningen, The Netherlands). After a warm-up period of 5 min of pedaling at 60 rpm interspersed with an all-out sprint lasting 5 s, the subjects pedaled for 30 sec at maximal speed against a constant force (1.0 Nm·kg^-1^). Peak power, mean power, time to peak power, total work and a fatigue index were determined. Peak power was defined as the highest mechanical power output elicited during the test. Mean power was defined as the average mechanical power during the 30 sec test. Fatigue index was determined by dividing the highest power output by the lowest power output.

#### Questionnaires

Subjects were instructed to assess their subjective feelings of energy, fatigue, alertness, and focus using a 15 cm visual analog scale (VAS). The VAS was assessed immediately before commencing each reaction test. Subjects were asked to assess via a mark on a 15-cm straight line, with words anchored at each end of the line, their feelings at that time. Questions were structured as "My level of focus is:" with low and high serving as the verbal anchor representing the extreme ratings. Similarly, "My level of energy is:" was anchored with the verbal cues "low" and "high", and while "My level of fatigue:" was anchored with the verbal cues "high" and "low". For fatigue, a higher score indicated greater fatigue. The validity and reliability of VAS in assessing fatigue and energy has been previously established [23]

#### Supplement

On the initial visit subjects consumed one serving (3 capsules) of either the supplement or placebo. Each serving of CRAM consisted of α-glycerophosphocholine (150 mg), choline bitartrate (125 mg), phosphatidylserine (50 mg), niacin (vitamin B3; 30 mg), pyridoxine HCl (vitamin B6; 30 mg), methylcobalamin (vitamin B12; 0.06 mg), folic acid (4 mg), L-tyrosine (500 mg), anhydrous caffeine (60 mg), acetyl-L-carnitine (500 mg), and naringin (20 mg). The placebo was similar in appearance to the supplement, but contained only an inert substance (rice flour). Subjects ingested the capsules with 12 ounces of bottled water.

#### Statistical Analyses

Statistical analysis of the data was accomplished using a 2 × 2 (time × treatment) mixed factorial analysis of variance. In the event of a significant F-ratio, Tukey post-hoc tests were used for pairwise comparisons. Chi-square analysis was used to compare responses between CRAM and PL groups on the yes/no survey questions. A criterion alpha level of p ≤ 0.05 was used to determine statistical significance. All data are reported as mean ± SD.

## Results

**Figure 2:**
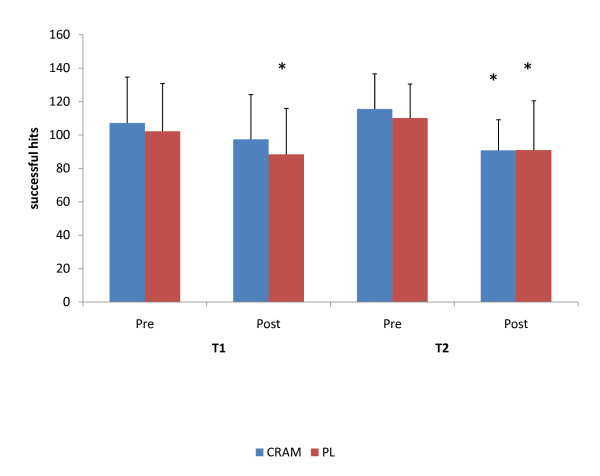
**Acute and Prolonged Effects of αGPC supplementation on Reaction Performance**. * = significantly different that Pre.

The effect of both acute and prolonged ingestion of the supplement on reaction time performance is depicted in Figure 2. Subjects consuming the supplement at T1 were able to maintain (p = 0.114) reaction time performance between PRE and POST measures, while a significant reduction (p = 0.050) between PRE and POST measures was observed in subjects consuming the placebo. However, no significant differences (F = 0.344, p = 0.565) were seen between the groups at either PRE or POST. Interestingly, both groups experienced significant declines from PRE to POST in reaction performance at T2. No significant differences (F = 0.235, p = 0.634) between the groups were seen in either PRE or POST following 4-weeks of supplementation. No significant differences in power or muscular endurance performance measures were seen between CRAM and PL groups at any time point (see Table 1).

**Table 1:** Acute and Prolonged Effects of CRAM supplementation on Power and Muscle Endurance Performance

		PP (W)	MP (W)	**PP (W·kg**^**-1**^**)**	**MP (W·kg**^**-1**^**)**	TW (J)	**Fatigue (W·s**^**-1**^**)**	Push-ups	Sit-ups
CRAM	T1	971 ± 119	621 ± 40	11.6 ± 1.5	7.4 ± 0.9	18627 ± 1189	20.5 ± 4.2	44.6 ± 12.6	33.1 ± 9.3
	
	T2	1009 ± 139	611 ± 40	12.7 ± 0.9	7.8 ± 0.7	18340 ± 1184	25.0 ± 7.2	43.4 ± 14.4	34.3 ± 9.2

PL	T1	1018 ± 307	633 ± 140	12.6 ± 2.0	7.5 ± 1.5	18174 ± 2875	25.6 ± 10.9	42.5 ± 10.8	35.3 ± 12.7
	
	T2	1058 ± 317	603 ± 114	12.9 ± 2.6	7.7 ± 1.5	18083 ± 3419	23.8 ± 7.1	44.2 ± 10.9	37.7 ± 10.6

**Figure 3:**
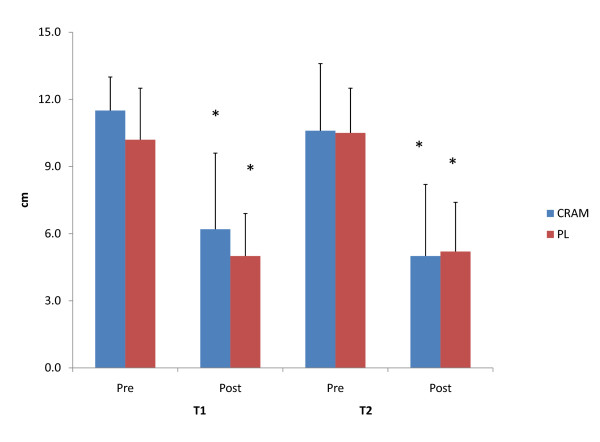
**Subjective Feelings of Energy**. * = significantly different that Pre.

**Figure 4:**
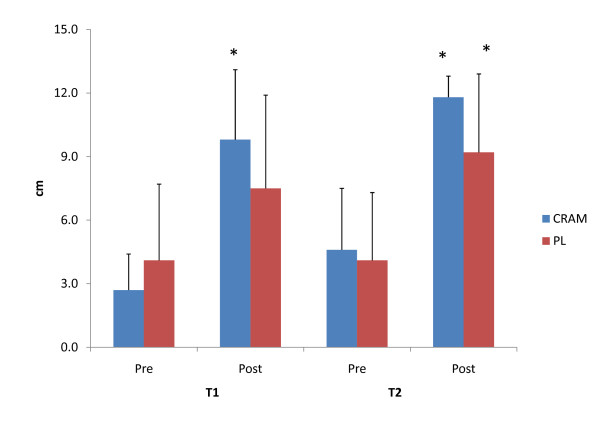
**Subjective Feelings of Fatigue**. * = significantly different that Pre.

**Figure 5:**
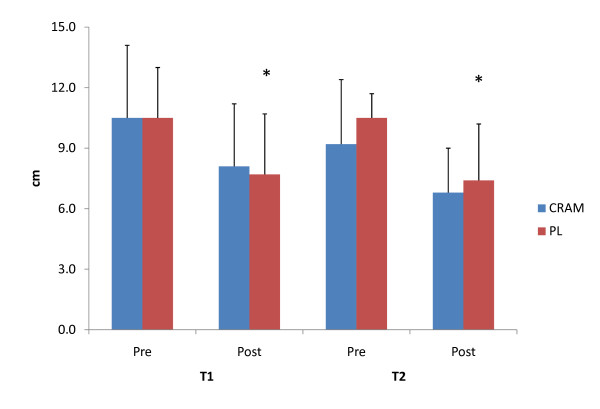
**Subjective Feelings of Focus**. * = significantly different that Pre.

**Figure 6:**
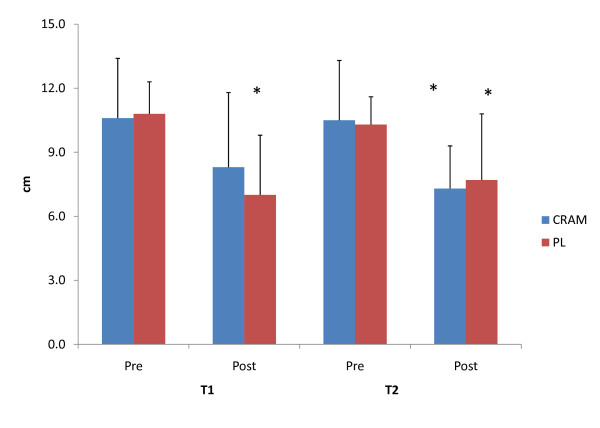
**Subjective Feelings of Alertness**. * = significantly different that Pre.

Subjective feelings of energy, fatigue, focus and alertness measured via a VAS are depicted in Figure 3, Figure 4, Figure 5 and Figure 6, respectively. Significant declines in subjective feelings of energy were observed between PRE and POST for both groups at T1 and T2. No significant differences in subjective measures of energy were seen between the groups at any time point. Elevations in subjective feelings of fatigue were seen for CRAM at both T1 (p = 0.001) and T2 (p = 0.000), but significant elevations in fatigue were seen at T2 (p = 0.029) only for PL. No differences were noted in fatigue levels between CRAM and PL groups at any time point. Subjects in the CRAM group were able to maintain their focus between PRE and POST during both T1 (p = 0.152) and T2 (p = 0.082) trials, whereas significant declines in focus were observed between PRE and POST in the PL group at T1 (p = 0.037) and T2 (p = 0.014). However, no differences in focus were seen between the groups at any time point. No differences between PRE and POST for subjective feelings of alertness were seen in the CRAM group at T1 (p = 0.83), but a significant decline in alertness was recorded at T2 (p = 0.040). Lower subjective levels of alertness were recorded at POST for T1 (p = 0.005) and T2 (p = 0.033) for the PL group. No differences in alertness though were seen between the groups at any time point.

## Discussion

Results of this study indicated that acute ingestion of CRAM can maintain reaction time to both visual and auditory stimuli following a high-intensity bout of exhaustive exercise, while subjects consuming a placebo experienced significant reductions in performance. In addition, acute ingestion of CRAM resulted in maintained focus and alertness following exhaustive exercise, while subjects consuming a placebo experienced significant declines in focus and alertness. Following 4 weeks of supplementation both groups exhibited significant declines in reaction performance. However, subjects consuming CRAM were still able to maintain their focus following exhaustive exercise, while subjects consuming a placebo did not.

Previous investigators have suggested that choline supplementation may provide an ergogenic benefit during prolonged or exhaustive exercise [1,7,8]. This has been based primarily on the assumption that exhaustive exercise may lower acetylcholine concentrations resulting in fatigue and lowered exercise performance. The basis of choline supplementation is that free choline can increase the rate of acetylcholine synthesis [24,25]. If acetylcholine levels become reduced during exhaustive exercise, supplementing with choline may maintain neurotransmitter concentrations and reduce fatigue and maintain performance. However, Spector and colleagues [26] reported that exercising until exhaustion at 70% of VO_2_max did not deplete choline. This is consistent with other studies reporting that choline concentrations may not be depleted during prolonged exercise [9,10], but contrasts with other studies showing reduced plasma choline concentrations during prolonged exercise [7,27,28]. Differences between these studies are difficult to explain considering that endurance exercise was the mode examined in these investigations, and subject populations were both recreationally and competitively-trained individuals. More consistent findings have been reported in choline's ability to enhance cognition and memory [5,7,29]. However, reports of enhanced memory or cognition following choline supplementation following a physical stress are limited. Only one study examined choline's potential to enhance cognitive performance following a physical stress, and results did not prove to be efficacious [9]. To date, it appears that the benefit of choline supplementation is inconclusive.

In contrast to the majority of research on choline ingestion, the present study incorporated relatively short-duration, high intensity anaerobic exercise protocol to elicit fatigue. Furthermore, the supplement ingested contained smaller concentrations of choline than has been previously shown to be efficacious. Despite these differences, the combination of other dietary ingredients appeared to have provided a positive effect on performance and subjective feelings of fatigue and alertness. To maximize the effectiveness of a supplement many sport nutrition companies combine several ingredients to provide a synergistic effect. The CRAM supplement combined choline (as α-glycerophosphocholine and choline bitartrate) with phosphatidylserine, carnitine, an energy matrix (caffeine and tyrosine) and vitamins. Phosphatidylserine has been previously shown to enhance recovery following high- and moderate-intensity exercise [1,15,20-22]. In addition, phosphatidylserine has been shown to enhance subjective feelings of energy, elation and confidence in healthy students subjected to stressful mental tasks [30] and in combination with carbohydrates to improve performance in golfers during induced stress [31]. Carnitine supplementation has been shown to enhance recovery following high intensity exercise [32,33], as reflected by reduced markers of muscle damage and a greater anabolic response (elevation in IGF binding protein) to exercise recovery. Although the vitamins included in CRAM are not known to be ergogenic when no deficiencies are present, the energy matrix found in this supplement has been shown to be effective in delaying time to fatigue and increasing volume of training [34].

The ability to maintain reaction performance following fatigue may have been due to the combined effect of choline, phosphatidylserine and the energy matrix. Although this is the first investigation to examine this combination of ingredients following exhaustive anaerobic exercise, previous studies have shown that this combination of ingredients to be effective in augmenting exercise [35] and cognitive [36] performance in rodents. Although the mechanism of action has not been fully elucidated, it has been suggested that this combination of ingredients may contribute to an enhanced neuroprotective effect via a stronger defense of membrane integrity [36]. Glycerophosphocholine and phosphatidylserine have been shown to form membrane phospholipids [37], and acetyl-L-carnitine may provide neuroprotective effects by buffering oxidative stress and maintaining energy supply to neurons [38]. The concentrations of ingredients used in CRAM appear to have been sufficient to maintain performance during T1; however, did not appear to provide the same effect at T2. This may have been due to habituation in that the daily concentration of ingredients ingested may not have provided the same physiological effect following 4 weeks of supplementation. Another potential explanation is that the weekly familiarization sessions that continued throughout the experimental period may have provided a training effect thereby making it more difficult for CRAM to affect performance at the same concentrations. However, the use of weekly familiarization sessions was critical to our study design to limit potential detraining effects. Thus, future research should address the role of chronic CRAM supplementation on acute exercise performance.

Despite the habituation effect observed for reaction time and subjective feelings of alertness, subjects' subjective feelings of focus in CRAM was maintained following the bout of high intensity exercise while subjects in PL experienced a significant decline. In conclusion, the results of this study indicate that acute ingestion of CRAM can prevent the exercise-induced decline of reaction time, and subjective feelings of focus and alertness in healthy college students following exhaustive exercise. However, some habituation may occur following 4-weeks of supplementation. Future investigations appear warranted to provide further insight on the efficacy of long-term supplementation of CRAM.

## Competing interests

JRH, NAR, AG, NAB, MWH, RJ and MP declare that they have no competing interests. MO is the CEO of MRM.

## Authors' contributions

JRH was the primary investigator, designed study, supervised all study recruitment, data/specimen analysis, statistical analysis and manuscript preparation. NAR was a co-authors, oversaw all aspects of study including recruitment, data/specimen analysis, and manuscript preparation. AG, NAB and MWH were co-authors, assisting with data collection and data analysis. RJ, MP and MO contributed to the conception and design of the study. RJ helped drafting the drafting the manuscript. All authors read and approved the final manuscript.
